# Presymptomatic Treatment with Acetylcholinesterase Antisense Oligonucleotides Prolongs Survival in ALS (G93A-SOD1) Mice

**DOI:** 10.1155/2013/845345

**Published:** 2013-12-22

**Authors:** Gotkine Marc, Rozenstein Leah, Einstein Ofira, Abramsky Oded, Argov Zohar, Rosenmann Hanna

**Affiliations:** Agnes Ginges Center for Neurogenetics, Department of Neurology, Hebrew University-Hadassah Medical Center, P.O. Box 12000, 91120 Jerusalem, Israel

## Abstract

*Objective*. Previous research suggests that acetylcholinesterase (AChE) may be involved in ALS pathogenesis. AChE enzyme inhibitors can upregulate AChE transcription which in certain contexts can have deleterious (noncatalytic) effects, making them theoretically harmful in ALS, whilst AChE antisense-oligonucleotides (mEN101), which downregulate AChE may be beneficial. Our aim was to investigate whether downregulation of AChE using mEN101 is beneficial in an ALS mouse model. *Methods*. ALS (G93A-SOD1) mice received saline, mEN101, inverse-EN101, or neostigmine. Treatments were administered from 5 weeks. Disease-onset and survival were recorded. Additional mice were sacrificed for pathological analysis at 15 weeks of age. In a follow-up experiment treatment was started at the symptomatic stage at a higher dose. *Results*. mEN101 given at the presymptomatic (but not symptomatic) stage prolonged survival and attenuated motor-neuron loss in ALS mice. In contrast, neostigmine exacerbated the clinical parameters. *Conclusions*. These results suggest that AChE may be involved in ALS pathogenesis. The accelerated disease course with neostigmine suggests that any beneficial effects of mEN101 occur through a non-catalytic rather than cholinergic mechanism.

## 1. Background

The mechanisms leading to cell death in amyotrophic lateral sclerosis (ALS) are not fully understood; oxidative stress, excitatory amino acids (EAAs), and apoptosis have all been implicated [1, 2].

Acetylcholinesterase (AChE), an enzyme primarily functioning in cholinergic synapses both in the central and peripheral nervous system, has been linked to processes or events occurring in ALS, namely, motor unit denervation [3] and EAA-mediated neurotoxicity [2, 4]. Suggestion of a role in denervation comes from experiments on transgenic (Tg) mice overexpressing human AChE which display abnormalities in neuromuscular structure similar to those observed in chronic denervation with reinnervation that is, motor-unit enlargement and neuromuscular-junction (NMJ) loss [3]. EAA-mediated overstimulation of motor neurons (MNs) results in secretion of AChE and this secretion precedes the neurotoxic effects induced by EAA [2, 4]. Furthermore, AChE has been shown to be present in tissues devoid of cholinergic synapses and to be involved in the process of apoptosis, a process also involved in ALS pathogenesis, by playing a pivotal role in apoptosome formation [5]. Clinical information is relatively sparse; elevated AChE levels were reported in the sera of ALS patients [6], as well as increased titers of IgG and IgA antibodies towards AChE [7]. Based on the circumstantial evidence linking AChE to ALS pathogenesis we sought to test the possibility that downregulation of AChE may be beneficial in an ALS mouse model. Because AChE may have noncatalytic effects [8, 9] together with the fact that AChE inhibition with drugs such as neostigmine (Neo) raise AChE levels [10], we chose to use antisense oligonucleotides (ASO) to AChE mRNA (mEN101) as our treatment drug. To address the possibility that mEN101's effect is mediated via a non-catalytic effect (i.e., reducing AChE levels) rather than via a decrease in catalytic activity, we compared mEN101 treatment to the AChE catalytic inhibitor Neo.

## 2. Materials and Methods

### 2.1. Mice

G93A-SOD1 (B6SJL-TgN[SOD1-G93A]1Gur) Tg mice [11] (Jackson-Laboratories, Bar-Harbor, ME) bred in the animal facility at Hadassah-Hebrew University Hospital were used. Experiments were approved by the Animal Ethics Committee.

### 2.2. Therapy Protocol

A total of 100 G93A-SOD1 Tg-mice were used for clinical studies, investigating the effect of early and long-term mEN101 (Ester Neurosciences) treatment. These studies included 2 experiments in which we compared mEN101 treatment ( 200 *μ*g /kg) with normal saline (NS) and a 3rd experiment, where mEN101 treatment was compared to NS as well as to an additional 2 control groups, namely, inverse sequence of mEN101 (Inv) (200 *μ*g/kg, Ester Neurosciences) and Neo (0.1 mg/kg, AstraZeneca). All mice received daily intraperitoneal injections of the appropriate treatment from 5 weeks until death. A total of 35 Tg-mice received mEN101, 35 NS, 15 Inv, and 15 Neo.

For pathological studies we used an additional 21 Tg-mice receiving either mEN101 (*n* = 7), NS (*n* = 9), Inv (*n* = 3), or Neo (*n* = 2), as well as in 12 wild-type (WT, non-Tg littermates) receiving NS, all of them sacrificed at 15 weeks of age.

In a follow-up experiment, a further 16 Tg-mice were used to investigate the effect of mEN101 treatment started at 12 weeks (when disease is clinically evident) at a higher dose (500 *μ*g/kg) compared to NS (*n* = 8/group).

### 2.3. Determination of Disease Onset and Survival

We followed mice by daily observation for survival and weekly testing of motor-function using a Rotarod device (Panlab, Barcelona) starting at 11 weeks of age. Onset of disease-related weakness was defined as a sustained decrease of more than 30% of baseline maximum running distance [12]. “Survival” was determined by an artificial endpoint: mice unable to right themselves 30 seconds after being placed on their sides were scored as “dead” and were sacrificed.

### 2.4. Spinal Cord Histology and Neuron Counts

For histopathological analysis, animals were anesthetized with a lethal dose of pentobarbital and subjected to perfusion via the ascending aorta with ice-cold PBS followed by 4% paraformaldehyde. The spinal cord tissues were dissected and postfixed in 4% paraformaldehyde for 24 hours.

For the quantification of the numbers of MNs, 5 *μ*m paraffin embedded transverse sections was stained with hematoxylin and eosin (H&E). Every tenth section (50 *μ*m gap between sections) in 2 different rostrocaudal levels of the lumbosacral spinal cord was examined by a light microscope at ×20 magnification under a grid overlay. Higher magnification (×40) was used when necessary. MNs were identified on the basis of morphological characteristics (large cells with single nucleolus located within the nucleus). The number of MNs per anterior horn (either left or right) of the spinal cord cross section was counted.

### 2.5. Statistical Analysis

Median survival analysis was performed by Kaplan-Meier analysis using SPS12 for windows. The unpaired *t*-test was used to compare mean MN counts between the study groups expressed as M ± SEM.

## 3. Results

**Figure 1:**
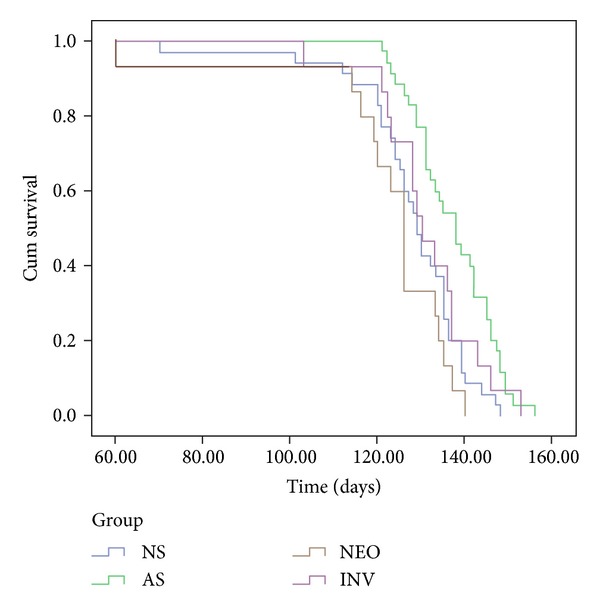
Presymptomatic mEN101 treatment delays death in the Tg-mice when compared to normal saline (NS), neostigmine (Neo), and inverse mEN101 (INV). Cumulative probability of death was delayed by mEN101. The mean time to death was significantly prolonged by 9 days in animals treated with mEN101 (*n* = 35) compared to NS (*n* = 35) (*P* < 0.001) and by 15 days when compared to Neo (*n* = 15) (*P* < 0.001). No significant effect on survival was found in the Inv (*n* = 15) or Neo (*n* = 15) treated groups when compared to NS although a nonsignificant trend towards earlier death was seen in the Neo group.

In all 3 experiments testing early treatment, a similar beneficial effect of mEN101 relative to NS was noted on the clinical parameters; thus the data was pooled, showing that mEN101 (200 *μ*g/kg, from 5 weeks of age) resulted in a nonsignificant 5-day delay in onset (from 114 to 119 days, *P* > 0.1) and a significant 9-day delay in death of the Tg-mice relative to NS treated mice (from 128 days to 137 days, *P* < 0.001). Treatment with Inv showed no change in age at onset (115 days) and death (131 days) relative to NS treated, while Neo treatment resulted in an earlier onset by 5 days (114 versus 109 days *P* < 0.05) and a nonsignificant decrease of 6 days in survival compared to the NS group (128 versus 122 days *P* > 0.05). The effect of mEN101 was significantly superior to that of Neo: a delay of 10 days to onset (119 versus 109 days *P* < 0.05) and 15 days to death (122 versus 137 days *P* < 0.001) (Figure 1). When treatment was started at 12 weeks at a higher dose (500 *μ*g/kg), the survival was not significantly prolonged with mEN101 when compared to NS (127 versus 123, *P* > 0.05).

**Figure 2:**
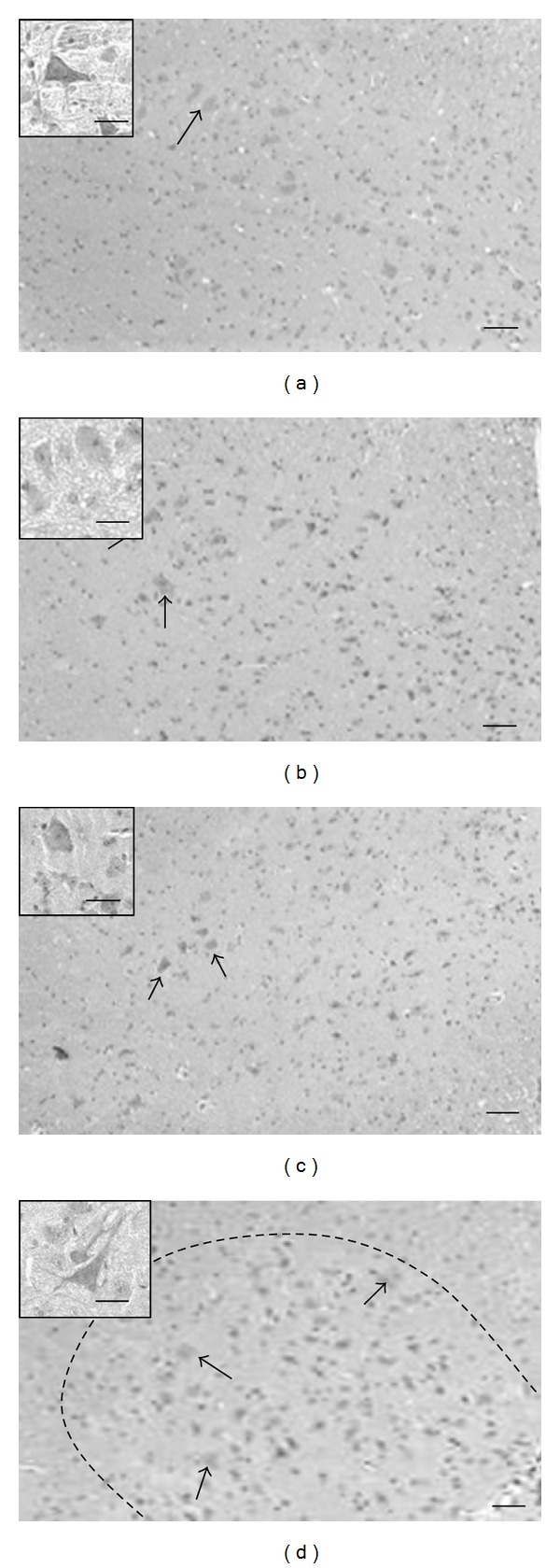
Pre-symptomatic mEN101 treatment attenuates motor neuron death in the Tg-mice when compared to normal saline. At 15 weeks of age, mEN101 treated Tg-mice (*n* = 7) (b) had significantly higher MN counts/anterior horn section compared to the NS treated Tg-group (*n* = 5) (a) (*P* < 0.0002). A small group of Inv treated Tg-mice (c) also had higher MN counts (*n* = 3). The NS treated Tg-group (*n* = 5) (a) showed significantly lower MN counts than the NS treated WT mice (*n* = 12) (d) (*P* < 0.0001). Quantification results are presented in text. Dashed line in (d) indicates borders of the ventral horn. Magnification of MN in the ventral horn arrows indicate MNs. Scale bars = 50 *μ*M.

To detect a possible neuroprotective effect of the mEN101 treatment, we analyzed MN counts in the lumbosacral anterior horn at 15 weeks, a time when MN loss is evident in this model [11], and compared this data between study groups. As expected, MNs counts were significantly lower in the Tg-mice relative to the WT mice (4.6 ± 1.0 versus 19.2 ± 1.6 MN/anterior horn, *P* < 0.0001, Figures 2(a) and 2(d)). Comparing the MN counts in the mEN101 treated Tg-mice (*n* = 7, Figure 2(b)) with those in NS treated Tg-mice (*n* = 5, Figure 2(a)) revealed significantly higher MN counts in the mEN101 group (10.1 ± 0.91 versus 4.6 ± 1.0 MN/anterior horn, *P* < 0.0002). Surprisingly, a higher MN count was also seen in mice that received Inv (9.2 ± 0.6 MN/anterior horn), yet the sample size was too small to draw statistical conclusions (*n* = 3, Figure 2(c)). No change in MN count was noticed in the Neo treated mice (*n* = 2) compared to NS treated mice.

## 4. Discussion

Here we demonstrate that pre-symptomatic antisense treatment targeted at inhibition of AChE expression improved survival with a trend towards delay of disease onset and attenuated loss of motor neurons in Tg-mice. The effect on survival was only observed with pre-symptomatic administration, a fact which potentially limits clinical therapeutic application of these results. Nevertheless, these data support the role of AChE in ALS pathogenesis, consistent with previous research linking AChE to neurodegeneration [3–7, 13–16].

Anecdotal reports of transient disturbances in NMJ transmission have led to the occasional use of AChE enzyme inhibitors in ALS patients despite the lack of evidence of an effect in a controlled trial [17]. In theory the improvement in clinical parameters with mEN101 could have been due to a symptomatic benefit on transmission at the NMJ. Given that treatment with Neo actually worsened the clinical parameters in our study, this possibility alone is unlikely. Furthermore, the beneficial effect we observed with mEN101 in contrast to the detrimental effects of Neo suggests the beneficial effect of mEN101 is mediated via a non-catalytic mechanism and not simply through improved NMJ transmission as a consequence of attenuated AChE hydrolysis.

The Neo dose selection was based on previous reports indicating short-term safety in rodents [18]. Nevertheless it is possible that chronic cholinergic overactivity adversely affected the mice at this dose, whereas a lower dose would have had a different effect. The possibility that Neo might be harmful in ALS demands further studies; nevertheless these preliminary results suggest that clinicians should reconsider the practice of performing a symptomatic trial of AChE enzyme inhibitors in patients with ALS.

Although Inv did not improve the clinical parameters, there was an unexpected effect on MN counts that was similar to mEN101. Although Inv is not expected to act through classical antisense mechanisms on mRNA transcription, the possibility that ASOs often act through nonantisense mechanisms has been supported [19]. More specifically, the beneficial effect of the humanized form of mEN101 in attenuating inflammatory responses in Sjogren's syndrome [20] and in models of posttraumatic anxiety [21] has been attributed to noncanonical activation of NF-*κ*B via activation of Toll-like-receptor 9. Shifting the balance away from canonical and towards noncanonical NF-*κ*B activation is an attractive putative mechanism for the beneficial effects of this antisense therapy in ALS, as p65 NF-*κ*B (which increases with canonical pathway activation), has been strongly implicated in the pathogenesis of ALS through interaction with TDP-43 [22]. Furthermore, EN101 reduces markers of inflammation such as IL-1*β* and IL-6 in primate motoneurons, which provides additional support for an anti-inflammatory mechanism of the antisense therapy [23]. In order to assess the possible contribution of anti-inflammatory mechanisms, future studies could include immunohistochemical analysis of inflammatory cytokines in mEN101-treated ALS model systems. Despite the evidence supporting nonantisense mechanisms, the superior clinical effect of mEN101 compared to Inv does support the theory that the specificity of the ASO is important and may point that mEN101 possesses a specific anti-AChE effect. Additional studies using a wider range of antisense controls may be needed to elucidate this issue.

To conclude, this preliminary study further supports the involvement of AChE in ALS-pathogenesis, while raising the possibility of an exacerbating effect of AChE enzyme inhibitors in this disease. Future studies should be directed at confirming the effect of mEN101 in both the G93A mouse model as well as other model systems and delineating the mechanisms of this effect.
